# Machine learning-enhanced gas sensor technology identifies ovarian and endometrial cancer of all stages through plasma volatile organic compound patterns

**DOI:** 10.1016/j.ebiom.2025.106027

**Published:** 2025-11-13

**Authors:** Jens Eriksson, Donatella Puglisi, Filip Herbst, Arturas Dobilas, Ivan Shtepliuk, Ulrika Joneborg, Henrik Falconer, Angelique Flöter Rådestad, Christer Borgfeldt

**Affiliations:** a Department of Physics, Chemistry and Biology (IFM), Linköping University, Sweden; b VOC Diagnostics AB, LEAD Linköping AB Teknikringen 7, Sweden; c Department of Obstetrics and Gynaecology, Skåne University Hospital (SUS), Lund University, Sweden; d Department of Women's and Children's Health, Karolinska Institute, Department of Pelvic Cancer, Theme Cancer, Karolinska University Hospital, Stockholm, Sweden; e Department of Women's and Children's Health, Karolinska Institute, Department of Hereditary Cancer, Theme Cancer, Karolinska University Hospital and Stockholm, Sweden; f Department of Obstetrics and Gynaecology, Department of Biomedical and Clinical Sciences, Linköping University, Linköping, Sweden

**Keywords:** Ovarian cancer, Endometrial cancer, Plasma analysis, Volatile organic compounds, Electronic nose, Metabolomics

## Abstract

**Background:**

Ovarian cancer presents with non-specific symptoms that make early diagnosis challenging and the prognosis poor. Ovarian and endometrial cancers exhibit similar genomic mutations and biomarker profiles. Endogenous volatile organic compounds (VOCs) are products of metabolic activity. In cancer, metabolites increase due to tumour necrosis, leading to cancer-specific VOC patterns. The aim of this study was to evaluate VOC analyses in plasma as diagnostic tests for early diagnosis of ovarian and endometrial cancer.

**Methods:**

Preoperative plasma from 133 women with ovarian cancer (stages I–IV or borderline ovarian tumors) and 41 women with endometrial cancer (stages I-IV) was compared to 115 healthy controls with highly sensitive gas sensors. Data analyses were performed using feature extraction from 32 gas sensors per sample. 85 features were extracted from each signal (including statistical, time-domain, and frequency-domain features), and training and test datasets were formed. The features underwent an iterative redundancy removal process for stepwise optimization of models. Model robustness was assessed using a train/test split scheme with unique datasets, leading to a model optimized for diagnostic performance. By implementing sequential binary classification boosting-based models, it was possible to determine not only the presence or not of cancer, but also to distinguish between ovarian- and endometrial cancer, and the stage of the cancer.

**Findings:**

The VOC analysis, powered by a 5-fold cross-validated ensemble classifier, achieved exceptional diagnostic performance. It correctly identified all 133 patients with ovarian cancer and borderline ovarian tumors, all 41 cases of endometrial cancer, and all 115 healthy controls. For staging, the model accurately classified 172 out of 174 (98.9%) cancer cases as stage I vs. II–IV. On validation data, the classifier yielded an accuracy of 96.63% (95% CI: 96.56%–96.70%), sensitivity of 96.42% (95% CI: 96.29%–96.54%), and specificity of 96.88% (95% CI: 96.76%–97.01%). These metrics were robustly replicated on the independent test set, with an accuracy of 97.13% (95% CI: 96.80%–97.45%), sensitivity of 96.92% (95% CI: 96.49%–97.35%), and specificity of 97.37% (95% CI: 96.97%–97.77%). Complementing this, four additional classifiers (each with accuracy exceeding 90%) were developed and integrated into a cascaded algorithm to enable multi-class discrimination (ovarian cancer and endometrial cancer vs. healthy controls), cancer typing (ovarian vs. endometrial), and staging (stage I vs. later stages). The analysis of VOCs correctly identified 133 out of 133 patients with ovarian cancer and borderline ovarian tumour. All 41 cases of endometrial cancer were correctly identified, as were all the 115 healthy controls. In 172 out of 174 (98.9%) cancer cases the model correctly classified stage I vs. II-IV.

**Interpretation:**

VOC analysis emitted to gas-phase from plasma demonstrates high sensitivity and specificity for diagnosing ovarian cancer, including borderline ovarian tumors and endometrial cancers, compared to healthy controls. VOC analyses accurately differentiated between early and advanced stages of both cancer types. Future external validation needs to be performed.

**Funding:**

The Strategic Innovation Programs Swelife and MedTech4Health, a joint venture of Vinnova, Formas and the Energy Agency (grant No. 2022-03464 and grant No. 2023-03874) and within the Convergence Accelerator Program (Track L – Real-World Chemical Sensing Applications), funded by the Swedish Research Council, Vetenskapsrådet (grant No. 2023-07219), and Sweden's Innovation Agency, Vinnova (grant No. 2023-04186), in collaboration with the US National Science Foundation (NSF). The computations, data handling, and machine learning model training and testing were conducted in the MATLAB environment and enabled by resources provided by the National Academic Infrastructure for Supercomputing in Sweden (NAISS), partially funded by the Swedish Research Council through grant agreement no. 2022–06725. This work received funding from the European Union's Horizon Europe Research and Innovation Programme under Grant Agreement No 101214318 (DISARM). Views and opinions expressed are however those of the author(s) only and do not necessarily reflect those of the European Union or the Health and Digital Executive Agency (HaDEA). Neither the European Union nor HaDEA can be held responsible for them.

**Research in Context**

**Evidence before this study**

Ovarian cancer is often diagnosed at a late stage due to vague symptoms, contributing to poor prognosis. Endometrial cancer shares molecular features with ovarian cancer, including similar genomic mutations and biomarker profiles. Volatile organic compounds (VOCs), which are by-products of metabolic processes, have shown promise as non-invasive biomarkers in various cancers. However, few studies have explored VOCs in plasma for early detection of gynaecological cancers using advanced sensor technologies.

**Added value of this study**

This study demonstrates that VOC analysis using highly sensitive gas sensors can accurately distinguish between healthy individuals and patients with ovarian or endometrial cancer, including early-stage disease. The machine learning models coupled with majority vote algorithm achieved 100% sensitivity and specificity in this internal cohort and could also differentiate between cancer types and stages. This is the first study to report such high diagnostic performance using plasma VOCs for both ovarian and endometrial cancers.

**Implications of all the available evidence**

These findings suggest that plasma VOC analysis could become a powerful, non-invasive diagnostic tool for early detection and classification of gynaecological cancers. If validated in external cohorts, this approach could significantly improve early diagnosis and treatment planning, potentially improving patient outcomes.

## Introduction

Ovarian cancer (ovarian-/tubal-/peritoneal epithelial cancer) is usually diagnosed in advanced stages due to non-specific symptoms. The histological subtype and cancer stage are key factors in patient survival. Survival rates are low due to the fact that 60–75% of patients are diagnosed at late stages.^1^ The advanced stage (III–IV) at which ovarian cancer is frequently diagnosed substantially contributes to its high mortality rate, elevated treatment costs, and significant burden on patients. Numerous studies have been carried out to enhance early diagnosis of ovarian cancer; nevertheless, there is no single analysis that possesses both high sensitivity and high specificity with a reasonable cost in daily clinical practice. Ovarian and endometrial cancers, owing to their anatomical proximity and shared molecular characteristics, pose significant diagnostic challenges, particularly in patients presenting with subtle or non-specific symptoms, even though endometrial cancer often manifests with overt signs such as abnormal vaginal bleeding. Pre-operative staging can be assessed using CT or MRI scans; however, the definitive stage is typically determined through histopathological evaluation after surgical staging.

In the UK, the prevalence of a newly diagnosed ovarian cancer in primary care has been estimated as low as 0.023%.^2^^,^^3^ However, the prevalence of ovarian cancer in secondary referral healthcare units is around 10% when patients are referred due to clinical symptoms such as bloating, early satiety, abdominal pain, or urinary urgency. In these settings, gynaecologists use positive results from cancer antigen 125 (CA125) ELISA tests and ultrasonography as diagnostic tools.^4^ In tertiary care settings, the prevalence of ovarian cancer in women undergoing surgery for unclear ovarian pathology is often above 30% after thorough pre-operative evaluation.^5^, ^6^, ^7^ These figures highlight an urgent need for a non-invasive and simple test to detect ovarian cancer when non-specific symptoms are investigated in primary healthcare.

Metal oxide semiconductor (MOS)-based gas sensors have been demonstrated to respond to volatile organic compounds (VOCs) at low concentrations, which, in combination with signals generated from multiple sensors operating under different conditions (temperature, filters, sensing material) allows identification of disease specific volatilomes as statistical sensor signal patterns, making them particularly suitable for applications requiring high sensitivity in early disease diagnostics. Endogenous VOCs are products of metabolic activity in the body. Changes in the metabolism of these VOCs can be characteristic of specific disease processes such as cancer development.^8^ Analyses of VOCs emitted from plasma or urine, including triethylamine, pyridine, toluene, and other VOCs, using field-effect-transistor (FET)-based gas sensors have been demonstrated to accurately measure VOCs at low concentrations.^9^, ^10^, ^11^ The metabolism in cancer cells vastly differs from that of healthy cells. Elevated glycolysis leads to increases in lactate and fumarate metabolites resulting in altered VOC concentrations.^12^ Experimental data indicate that VOCs released by cancer cells may be detected in plasma enabling early diagnosis of cancer. In patients with ovarian cancer, analyses of specific VOCs in blood have shown promising outcomes in separating patients with ovarian cancer from healthy women.^13^^,^^14^ Analyses of VOCs in breath has shown to detect patients with lung, breast and head and neck cancer^15^, ^16^, ^17^, ^18^, ^19^ and in sweat VOCs differ between pre- and post-surgery status in patients with breast cancer.^20^ In urine, VOC biomarkers have been found detecting colorectal and lung cancer.^9^^,^^11^^,^^21^ Analyses of VOCs have also been studied for detecting other diseases, such as asthma, tuberculosis, cystic fibrosis, and pneumonia.^8^ Detection of cancer using VOCs is of great interest as it is a non-invasive and potentially inexpensive method. If the sensitivity for cancer detection proves high enough, VOC analyses may in the future be used in patients with non-specific symptoms or possibly also as screening method for ovarian cancer.

Given the overlapping molecular characteristics, ovarian and endometrial cancers warrant comparative investigation, particularly in light of the diagnostic challenges they pose—challenges that are compounded by the often subtle presentation of ovarian cancer and the typically more overt symptoms of endometrial cancer.^22^ The ability to accurately distinguish between these two malignancies is crucial, particularly in cases where diagnostic imaging results are ambiguous, potentially facilitating more precise and timely diagnoses. The imperative to develop a reliable method for differentiation is further underscored by the observation that even advanced cell free DNA profiling and protein biomarker analyses will fail to definitively identify the cancer types.^23^

This study explores the use of VOC analysis in plasma as a novel diagnostic tool for early detection of ovarian and endometrial cancers. We conducted a comparative analysis using preoperative plasma samples from patients with cancer and healthy controls, applying advanced gas sensor technology and machine learning to extract and optimize diagnostic features. The motivation stems from the urgent need for earlier and more accurate detection methods, given the non-specific symptoms and poor prognosis associated with ovarian cancer. Our findings demonstrate very high diagnostic accuracy, correctly identifying cancer cases and healthy controls, and effectively distinguishing between cancer types and stages. This work is important because it introduces a non-invasive, highly sensitive method that could transform early cancer detection and improve patient outcomes.

## Methods

### Study subjects

Peripheral blood samples (1 ml plasma) were obtained preoperatively from patients with suspicion of ovarian malignancy at Karolinska University Hospital 2019–2020 and Lund University Hospital 2016–2020, and endometrial cancer 2015–2022 and stored at −80 °C in biobanks until analyses. At Karolinska, patients were included in the study if they were suspected to have stage III or IV high-grade ovarian, fallopian tube, or peritoneal carcinoma. In the Lund cohort, all patients with suspected ovarian malignancies, as well as those with confirmed endometrial cancer, were consecutively enrolled in the biobank. From this biobank, we requested samples representing borderline ovarian tumors, as well as early-stage and advanced-stage ovarian and endometrial cancers, selected according to the morphological distribution present within the biobank. Women under 18 years of age or not able to sign informed consent were excluded. Plasma samples from patients diagnosed with epithelial borderline ovarian tumors, ovarian cancer, and endometrial cancer were analysed and compared to fresh frozen plasma stored at −80 °C, obtained from healthy blood donors at Linköping University Hospital. All patients were classified according to the ICD-10 (International Classification of Diseases, 10th Revision) on the location of the tumor and the FIGO (International Federation of Gynaecology and Obstetrics) staging system. The healthy blood donors, comprising women aged 30–60 years, had all provided informed consent for the use of their plasma in research purposes. All plasma were collected in EDTA tubes and subsequently centrifuged at 3000 revolutions per minute for 10 min, stored in cryo-tubes at −80 °C awaiting analysis. Upon arrival at the lab, the samples are stored at −80 °C until the day of the measurement. Samples are always measured within 30 min of thawing to room temperature, and the vials containing the samples were sealed until they are transferred to the instrument sample holder for immediate measurement.

### Power analysis

A power analysis was conducted to determine the minimum sample sizes required to validate the diagnostic performance of a binary classification model, with a sensitivity of 97% and a specificity of 98% in distinguishing patients with cancer from healthy controls based on plasma biomarkers. Using a one-sample proportion test with a significance level (α) of 0.05 and a statistical power of 90%, the required sample sizes indicated minimum in total 23 samples (12 patient and 11 control samples).

### Volatile organic compound analyses

**Fig. 1:**
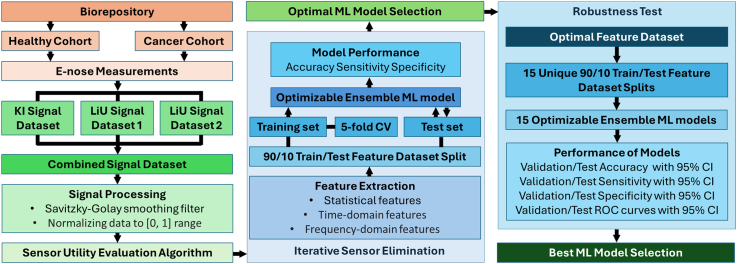
Flowchart. Multi-step framework for classifying blood samples using electronic nose technology and machine learning (ML), encompassing data acquisition, signal processing, sensor utility evaluation, feature extraction, model optimization, and stability assessment.

A multi-step approach was used for the classification of blood samples from healthy controls (Healthy Cohort) and samples from patients with ovarian or endometrial cancer (Cancer Cohort) by integrating electronic nose technology and machine learning (ML), see Fig. 1 Flowchart. The same approach was also used for staging of both cancer types. In the first step, blood samples from two categories were obtained from the biorepository. These blood samples were then analysed using an electronic nose, specifically developed by VOC Diagnostics AB (Linköping, Sweden) which incorporates 32 sensor elements (MOS-based sensors) designed to detect volatile organic compounds naturally emitted from blood samples at room temperature. Each sensor's response was recorded as a voltage-time signal over 600 s. Gas sensors (Figaro TGS2X series: TGS2602, TGS2603, TGS2620, TGS2611E, TGS2600, TGS2611C, TGS2444, TGS2610) (FIGARO INC. IL, USA) were configured in four banks with eight sensors each, where the four banks were operated at different temperatures in the range 200–400 °C. For each measurement, 1 ml of blood plasma was placed into a sample holder which was inserted into the e-nose sensor tunnel at the start of a measurement. After 40 s, a fan downstream of the sensor tunnel was started to transport the VOCs emitted from the blood plasma to the sensors. The sample and holder were removed after 360 s, and after 500 s the fan was switched off to allow for sensor recovery before the next measurement.

### Statistics

All data processing, feature extraction, and model training were performed using MATLAB Version 24.1.0.2537033 (R2024a). No additional external libraries beyond MATLAB's standard toolboxes (e.g., Signal Processing Toolbox, Statistics and Machine Learning Toolbox) were required, and all toolboxes were compatible with MATLAB R2024a. The measurements were conducted in two different places and in three different patient batches—Karolinska Institute (KI) batch 1 and Linköping University (LiU) batch 2 and 3 (LiU batch 2 and 3 with patient samples from Lund University biobank). This step ensured broader validation of results and reduced the potential for lab-specific biases. Consequently, three separate signal datasets were generated, which were then combined into a final signal dataset. All details related to signal processing are provided in Section S1, Signal Processing (Supplementary Material).

Before proceeding to feature extraction, we posed the question: do all sensors exhibit the same discriminatory power, i.e., the ability to differentiate signals recorded in the presence of blood samples from different categories? Evidently, data (signals) from sensors with low discriminatory power would be nearly identical and, thus, of limited value, making them candidates for exclusion. To address this, we developed a sensor utility evaluation algorithm based on calculating the average Pearson correlation coefficient (also called the similarity coefficient) for all pairs of signals generated by a given sensor for samples from the two categories. Sensors with low discriminatory power showed a high correlation coefficient, and vice versa. By calculating the similarity coefficient for all 32 sensors, we ranked the sensors by their utility. Next, we developed an iterative sensor elimination algorithm using a for-loop structure (for more details please see Section 2, Supplementary Material).

As a result, we trained and tested 32 Optimizable Ensemble models, generating a performance matrix (visualization of this matrix is given in Fig. S1, Supplementary Material). By analyzing this matrix and considering the dataset size at each step, we identified the most advantageous combination of sensors to create an optimal machine learning model (balancing performance metrics and the size of the training dataset). Subsequently, we assessed the stability of this model. The idea was as follows: we applied the 90/10 train/test split Scheme 15 times to the feature dataset on which the optimal model was built. Each split was unique due to its random nature, meaning we created 15 unique training and 15 unique test datasets. Clearly, a reliable model should not depend on the randomness of the feature dataset split. Ultimately, we obtained performance parameters for 15 ML models, identified the best-performing model (with the highest robustness), and saved it as the final classifier model.

**Fig. 2:**
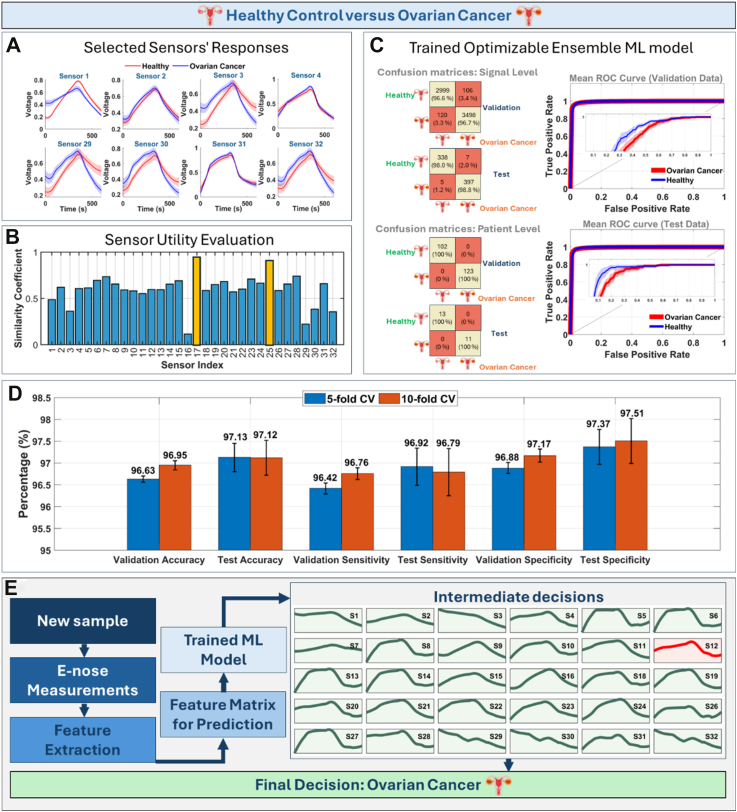
Overview of the development, performance evaluation, and application of the ML model for distinguishing blood samples from 115 healthy women and 133 samples from women diagnosed with ovarian cancer (with one ovarian cancer sample measured in duplicate, resulting in 134 signals total; all other samples measured once). (A) Mean signals (with 95% confidence intervals) generated by selected individual sensors in the presence of samples from the two categories, highlighting differences in sensitivity to VOC biomarkers. (B) Sensor utility ranking based on the sensor utility evaluation algorithm, identifying the least useful sensors (#17 and #25, highlighted in yellow). (C) Validation and test signal-level and patient-level confusion matrices and ROC curves demonstrating high classification accuracy (∼97%) after optimization. (D) Robustness test results showing minimal influence of training/test split randomness and CV scheme on model performance metrics. (E) Example of intermediate and final predictions for a patient-level diagnosis using the majority voting algorithm, ensuring robust classification outcomes. This panel demonstrates e-nose responses to OC sample across 30 sensors and model's intermediate predictions (correctly classified: light green background/dark green curve; incorrectly classified, e.g., Sensor #12: light red background/dark red curve). Final patient diagnosis: majority vote (≥90% sensor agreement).

In Fig. 2, the ML model, based on Gentle Adaptive Boosting, is further visualized. It was designed to distinguish blood samples from healthy women and those with ovarian cancer. Fig. 2A illustrates the signals generated by individual sensors in the presence of samples from the two categories. It is evident that some sensors, such as Sensor #2, Sensor #4, and Sensor #31, are less sensitive to ovarian cancer VOC biomarkers compared to others, as indicated by the similarity of signals across the two categories. The application of a sensor utility evaluation algorithm allowed us to rank the sensors based on their utility (Fig. 2B) and ultimately identify that excluding data from just two of the least useful sensors, #17 and #25 (highlighted in yellow), enabled high classification accuracy, as confirmed by the validation and test confusion matrices and ROC curves (Fig. 2C). Moreover, we conducted a robustness test (results shown in Fig. 2D) and found minimal influence from both the randomness of the training/test split process and the cross-validation scheme on model accuracy, specificity, and sensitivity, which consistently remained at approximately 97% for this specific sensor. It is important to note that the model is initially designed to classify signals generated by sensors for two categories of samples. This means that for a single patient, the model generates 30 intermediate predictions regarding their status. For instance, in the case of a 62-year-old woman with Low-Grade Serous Carcinoma (C56.9) in the ovary at Stage IIIC, we plotted the 30 signals generated by the e-nose for this sample (Fig. 2E), which were subsequently classified by our model (so-called intermediate decisions). Signals correctly classified as the “Ovarian cancer” category are visualized with a green background and curve colour, while those misclassified as “Healthy individual” appear in red. Notably, the signal from Sensor #12 fell into the latter category, highlighting a specific instance of model error. However, the final decision that the patient has ovarian cancer was made using a majority voting algorithm, which provided a definitive decision only if more than 90% (a predefined threshold we set) of the predictions for signal categorization aligned. In this case, 29 signals were correctly classified, ensuring an accurate final patient-level diagnosis. Returning to Fig. 2C, it is clear that the Bayesian-optimized GentleBoost ensemble (with an accuracy of approximately 97%) coupled with the majority voting algorithm produces ideal patient-level confusion matrices at both validation and test levels. It is worth noting that the same approach was applied to other classification tasks examined in this study, with the only difference being the unique ranking of sensor utility by the evaluation algorithm in each case, effectively demonstrating the reusability of the feature extraction pipeline.

Ultimately, by integrating data from patients with ovarian cancer, endometrial cancer, and healthy controls, we developed a cascade algorithm to determine the sample status. This algorithm incorporates four trained and tested Optimizable Ensemble ML models (Classifiers 1–4, which are based on Bayesian-optimized GentleBoost ensemble algorithm).

The process begins with Classifier 1, which determines whether the patient is in a state of cancer or not. If the outcome indicates no cancer, a final decision is made that the patient is healthy. If cancer is detected, the algorithm proceeds to Classifier 2, which identifies the specific cancer type as either ovarian or endometrial.

Based on this result, the algorithm advances to either Classifier 3 (to determine the stage of ovarian cancer: Stage I or Stages II–IV) or Classifier 4 (to determine the stage of endometrial cancer: Stage I or Stages II–IV).

To mitigate the risk of overfitting in our Optimizable Ensemble model, we implemented several strategies (model simplification, feature reduction, and constrained Bayesian optimization), as detailed in Supplementary Material Section S3. Fig. S2 presents the validation and test confusion matrices for all evaluated models (with optimized hyperparameters detailed in Table S1), including the optimal one. Complementing this, Fig. S3 provides a comparison of key performance metrics (accuracy, sensitivity, and specificity) across both validation and test sets. Notably, the various model manipulations, informed by the outlined strategies, yielded negligible impacts on classifier performance, underscoring the inherent robustness and architectural stability. This observation is further corroborated by the analysis of learning curves (Fig. S4), which exhibit minimal divergence between training, test, and validation trajectories, indicating minimal overfitting and strong generalization capabilities on held-out data.

### Ethics

Written informed consent was obtained from all study participants. Ethical approvals for the study were obtained from the Ethical Review Board at the Faculty of Medicine, Karolinska University Hospital (Dnr: 2019–02140) for patients recruited at Karolinska University Hospital, and from the Ethical Review Board at the Department of Medicine, Lund University (Dnr: 2022-03015-02 and amendment Dnr: 2023-01881-01) for patients recruited at Lund University Hospital.

### Role of funders

The funders were not involved in the design of the study, data collection, analysis, interpretation of results, or the writing of the manuscript. All aspects of the research were conducted independently by the authors.

## Results

Plasma samples from patients with ovarian cancer (*n* = 123) at stages I–IV and borderline patients with ovarian tumor (*n* = 10), aged 18–89 years, were compared with those from patients with endometrial cancer (*n* = 41), aged 41–90 years, as well as healthy controls (*n* = 115), aged 30–60 years (Table 1). The morphological cancer types and stages are detailed in Table 1.

**Table 1:** Patient characteristics.

Karolinska Hospital	Ovarian cancer n = 45					
Age (years)	Median	71	Range	48	–	89
	Mean	69	SD	10,1		
Lund University Hospital	Ovarian cancer n = 88					
Age (years)	Median	63	Range	26	–	86
	Mean	62	SD	12,6		
Lund University Hospital	Endometrial cancer n = 41					
Age (years)	Median	67	Range	41	–	90
	Mean	67	SD	10,6		
**Ovarian Cancer**	**Total**	133				
Stage I	Borderline Mucinous	2				
	Borderline Serous	5				
	Clear cell cancer	10				
	Endometrioid	9				
	High Grade serous	4				
	Low grade serou	2				
	Mucinous	8				
Stage II–IV	Borderline Serous	3				
	Clear cell cancer	10				
	Endometrioid	5				
	High Grade serous	63				
	Low grade serous	9				
	Mucinous	3				
**Endometrial Cancer**	**Total**	**41**				
Stage I	Endometrioid	14				
	Serous	2				
Stage II–IV	Clear cell cancer	3				
	Endometrioid	15				
	Serous	7				
**Healthy Controls**		**115**				

**Fig. 3:**
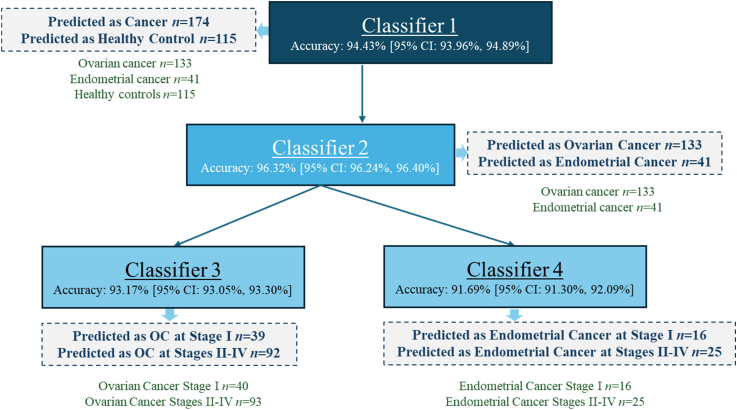
Flowchart illustrating the cascade machine learning algorithm for determining cancer type and stage, based on electronic nose measurements of VOCs emitted from blood plasma samples of 115 healthy controls, 133 OC patients, and 41 EC patients. Classifier 1 distinguishes healthy controls from patients with cancer (OC or EC). If cancer is detected, Classifier 2 differentiates between OC and EC. Depending on the Classifier 2 decision, Classifier 3 determines the stage (early vs. advanced) for OC patients. Classifier 4 determines the stage for EC patients. It is important to note that the accuracies reported for each classifier reflect the model's performance at the signal classification level. Meanwhile, the final predictions shown in the dashed rectangles were obtained using the majority voting algorithm. The green text below the dashed rectangles indicates the number of samples with true labels.

VOCs emitted from biosamples were measured using the e-nose system. A boosting ensemble machine learning model was developed to classify samples based on these measurements, generating 32 signal-level predictions per sample. Individual signal-level predictions achieved validation and test accuracy, sensitivity, and specificity below 97%. A majority-voting algorithm was applied, classifying a sample as cancerous or healthy only if at least 90% of the sensor predictions agreed on the same outcome. This approach correctly identified all patients with cancer as having cancer (*n* = 174) and all healthy controls as healthy (*n* = 115), as shown in Fig. 3. Consequently, the sensitivity and specificity at the sample level reached 100%, with no false negatives or false positives. Note that the reported 100% performance reflects the aggregated decision at the sample level, whereas individual signal-level predictions exhibited lower accuracy. In the second analysis, the VOC analyses successfully distinguished patients with ovarian cancer from patients with endometrial cancer without any misclassifications. In the third analysis, early-stage cancer (stage I) was differentiated from advanced stages (stage II–IV). Within the ovarian cancer cohort, one patient with stage II was incorrectly predicted as having early-stage (stage I) cancer, and one patient with early-stage ovarian cancer was incorrectly predicted as having advanced-stage (stage II–IV) cancer. For Stage I predictions, the sensitivity was 97% (95% confidence interval [CI]; 0.93–1.0), the specificity was 99% (95% CI; 0.97–1.0). The accuracy was 98% (95% CI) 0.96–1.0).

For Stage II-IV predictions, the sensitivity was 99% (95% CI; 0.97–1.0), the specificity was 97% (95% CI; 0.93–1.0). The accuracy was 98% (95% CI; 0.96–1.0). All the patients with endometrial cancer were classified in the correct stage leading to 100% sensitivity and specificity.

## Discussion

Previous studies have demonstrated that VOCs can be utilized for diagnostic purposes in detecting malignancies and other medical conditions through various biological samples such as plasma, sweat, breath, and urine.^8^^,^^20^^,^^24^^,^^25^ In our study, we have shown that VOC analysis in plasma can accurately differentiate patients with ovarian cancer, endometrial cancer (across all stages), and borderline ovarian tumors from healthy controls. Our findings demonstrate a sensitivity of 97% at the signal level and 100% at the sample level, outperforming the 92% sensitivity reported by Horvath et al. for ovarian cancer detection using a comparable approach.^12^

This study provides initial insights into the analysis of VOCs in patients with endometrial cancer. The method successfully distinguished all patients with endometrial cancer from healthy controls and patients with ovarian tumor. Additionally, our analyses were able to predict early-stage vs. advanced-stage ovarian cancers in all but two cases. In the case of endometrial cancer, all patients were accurately classified as either early or late stage.

When ovarian malignancy is suspected, CA125 is the most used tumor marker. However, CA125 has low sensitivity in early-stage ovarian cancer and low specificity as it can be elevated in benign diseases.^26^ CA125 is more sensitive in epithelial ovarian cancer than in other ovarian tumors.^27^ Human epididymis protein 4 (HE4), alone or with CA125 in the Risk of Ovarian Malignancy Algorithm (ROMA), increases sensitivity and specificity, especially in pre-menopausal women.^28^^,^^29^ HE4 is FDA-approved for monitoring epithelial ovarian cancer but is less widely used than CA125. Transvaginal ultrasonography (TVS) is standard for suspected adnexal lesions but is operator dependent. Morphological features and Doppler measurements in TVS are combined in various models. The Risk of Malignancy Index (RMI) uses TVS, menopausal status, and CA125, but has poor sensitivity in early-stage ovarian cancer.^30^ The International Ovarian Tumor Analysis (IOTA) recommends TVS-based models like Logistic Regression model 2 (LR2) and Assessment of Different NEoplasias in the adneXa model (ADNEX) for higher diagnostic accuracy.^6^^,^^31^ LR2 estimates malignancy probability using age and TVS findings, while ADNEX discriminates between benign, borderline, early, and advanced malignancies.^32^

ROMA, LR2, and ADNEX have better sensitivity than RMI in both pre- and postmenopausal women, but ROMA and ADNEX have decreased specificity.^33^ Other types of analyses have shown that a 'disease fingerprint' acquired via machine learning from the spectra of near-infrared fluorescence emissions of an array of carbon nanotubes functionalized with quantum defects detects high-grade serous ovarian carcinoma in serum samples from symptomatic individuals with high sensitivity and with very high (98%) specificity.^34^ Recently artificial intelligence (AI)-driven ultrasound support models have shown improved accuracy as diagnostic support.^35^ However, research frequently focuses on highly selected referral populations instead of general populations. In general practice, a straightforward blood test with high sensitivity for ovarian neoplasms, such as VOC analyses, would be advantageous for both patients and practitioners. Secondary analysis utilizing TVS, ROMA, ADNEX, or TVS with AI-supported models would be valuable. Future research should evaluate the efficacy of a combined VOC and ROMA model or TVS with AI-supported models in a primary care setting through clinical trials.

Finding endometrial cancer early is crucial because it significantly improves the chances of successful treatment with surgery without addition of chemotherapy or radiotherapy. Early detection means the cancer is still localized to the uterus and has not spread. If the lymph nodes include metastases adjuvant treatment is recommended. In our VOC analyses the patients with endometrial cancer were all accurately classified as either early or late stage. In widespread endometrial cancer there will be benefits for the clinician to know the stage of the cancer before surgery to better tailor the surgery of the patient.

Gas Chromatography – Mass Spectrometry (GC–MS) has been the gold standard technique of VOC analysis for decades. By providing both separation and identification of individual VOCs, it allows for precise qualitative and quantitative analysis of chemical compounds. However, this approach has limitations as it relies on sophisticated instrumentation that requires time-consuming and resource-intensive sample preparation and analysis, along with highly trained personnels, resulting in expensive analysis. An additional drawback of GC–MS is that high temperatures can degrade some VOCs into simpler, low-weight VOCs. This means GC–MS might detect degradation products instead of the actual VOCs, complicating the link between cancer and specific biomarkers. To increase efficacy and allow near-real time analyses without the need to separate and identify individual VOCs, the electronic nose approach based on highly sensitive gas sensors, as we use it, has been introduced. These sensors work based on changes in electrical properties induced by the interaction (adsorption) of VOCs with the sensor surface. Data analysis is empowered by machine-learning models. Nanomaterial-based sensors with crystal domains in the 10–100 nm range have been developed and applied in diagnostics of diseases with high sensitivity, speed of detection, and the ability to detect multiple targets. The sensors are designed to mimic the human olfactory system with VOCs adsorbing to a sensor array. The number of VOCs that can be detected and their types are different between human olfactory and the e-nose which can detect non-odorant gases.^36^

In this study, only epithelial ovarian cancer types and endometrial cancer were included. Excluding certain tumour types alters the population spectrum. The performance of the VOC analyses in other cancer types and non-epithelial ovarian histological subtypes need further analysis. Plasma samples from healthy controls were obtained from slightly younger blood donors and not from the same biobanks as the cancer samples, which presents a limitation. Differences in sample handling and age distribution may influence VOC composition. However, the analysis successfully distinguished between ovarian and endometrial cancer samples from the same biobank, as well as between early and advanced disease stages. This strongly suggests that the high diagnostic accuracy was not due to control sample handling. Nonetheless, these factors underscore the need for future validation to ensure robustness and reproducibility of the findings.

We included samples from two distinct cancer types to demonstrate the model's ability to differentiate between various cancers and between localized and metastatic disease, as these factors significantly influence treatment recommendations. While it is conceivable that other tumor characteristics, such as morphological types, could be integrated into the model, this would likely necessitate larger sample sizes and has not yet been explored. Future research will involve further testing to distinguish additional cancer types.

In summary, we found that analysis of VOCs emitted gases from plasma blood using an electronic nose combined with machine learning show very high sensitivity and specificity in pre-operatively distinguishing patients with ovarian cancer and endometrial cancer with stages I-IV, and borderline ovarian tumors from healthy controls. Our findings underscore the great potential of using electronic nose technology and machine learning as a powerful tool that can help physicians and healthcare professionals accelerate diagnosis and anticipate decisions. A limitation of this study is the lack of an independent external validation cohort. Although we implemented several strategies to prevent overfitting, these methods cannot fully replace external validation. Future studies with larger patient cohorts and other cancer types will be carried out for further testing and validation.

## Contributors

Jens Eriksson: Conceptualization, data curation, verified the underlying data, formal analysis, funding acquisition, investigation, methodology, resources, supervision, validation, writing – review & editing.

Donatella Puglisi: Conceptualization, funding acquisition, investigation, methodology, project administration, resources, validation, writing – review & editing.

Filip Herbst: Conceptualization, verified the underlying data, writing – original draft, review & editing.

Arturas Dobilas: Conceptualization and review.

Ivan Shtepliuk: Data curation, formal analysis, funding acquisition, investigation, methodology, software, validation, visualization, writing – review & editing.

Ulrika Joneborg: Conceptualization and review.

Henrik Falconer: Conceptualization and review.

Angelique Flöter Rådestad: Conceptualization, data curation, verified the underlying data, writing – review & editing.

Christer Borgfeldt: Conceptualization, methodology, verified the underlying data, funding acquisition, project administration, supervision, writing – original draft, review & editing.

All authors have read and approved the final version of the manuscript.

## Data sharing statement

Data collected for the study, including individual, deidentified, participant data and a data dictionary defining each field in the set, will be made available upon request to the corresponding author.

## Declaration of interests

This is an academic sponsored study with grants from Vinnova, Formas and the Energy Agency, grant No. 2022-03464 and grant No. 2023-03874, Vetenskapsrådet, grant No. 2023-07219, Sweden's Innovation Agency, Vinnova, grant No. 2023-04186, in collaboration with the US National Science Foundation (NSF), National Academic Infrastructure for Supercomputing in Sweden (NAISS), partially funded by the Swedish Research Council through grant agreement no. 2022–06725. Jens Eriksson is Chief Technical Officer (CTO) at VOC Diagnostics, Jens Eriksson holds stock in VOC diagnostics, Christer Borgfeldt is a member of the Advisory Board (unpaid) at VOC Diagnostics. All other authors declare no conflicts of interest.
