# Supplementary material for: Machine learning-enhanced gas sensor technology identifies ovarian and endometrial cancer of all stages through plasma volatile organic compound patterns
Source: eBioMedicine. 2025 Nov 13;122:106027. doi: 10.1016/j.ebiom.2025.106027 (PMC12661361; doi:10.1016/j.ebiom.2025.106027)
Supplement: Multimedia component 1 [file mmc1.docx]

**Supplementary Material**

**Machine Learning-Enhanced Gas Sensor Technology Accurately Identifies Ovarian and Endometrial Cancer of all Stages Through Plasma Volatile Organic Compound Patterns**

Authors: Jens Eriksson^1,2^, Donatella Puglisi^1^, Filip Herbst^3^, Arturas Dobilas^3^, Ivan Shtepliuk^1^, Ulrika Joneborg^4^, Henrik Falconer^4^, Angelique Flöter Rådestad^5^, Christer Borgfeldt^6^ *

^1^[Department of Physics, Chemistry and Biology (IFM)](https://liu.se/en/organisation/liu/ifm), Linköping University, Sweden

^2^VOC Diagnostics AB, LEAD Linköping AB Teknikringen 7

^3^Department of Obstetrics and Gynecology, Skåne University Hospital (SUS), Lund University, Sweden

^4^Department of Women's and Children's Health, Karolinska Institute, and Department of Pelvic Cancer, Theme Cancer, Karolinska University Hospital, Stockholm, Sweden

^5^Department of Women’s and Children’s Health, Karolinska Institute, Department of Hereditary Cancer, Theme Cancer, Karolinska University Hospital and Stockholm, Sweden

^6^Department of Obstetrics and Gynecology, and Department of Biomedical and Clinical Sciences, Linköping University, Linköping, Sweden

*Corresponding author:

Christer Borgfeldt [christer.borgfeldt@liu.se](mailto:christer.borgfeldt@liu.se)

Department of Obstetrics and Gynecology, and Department of Biomedical and Clinical Sciences, Linköping University, Linköping, Sweden


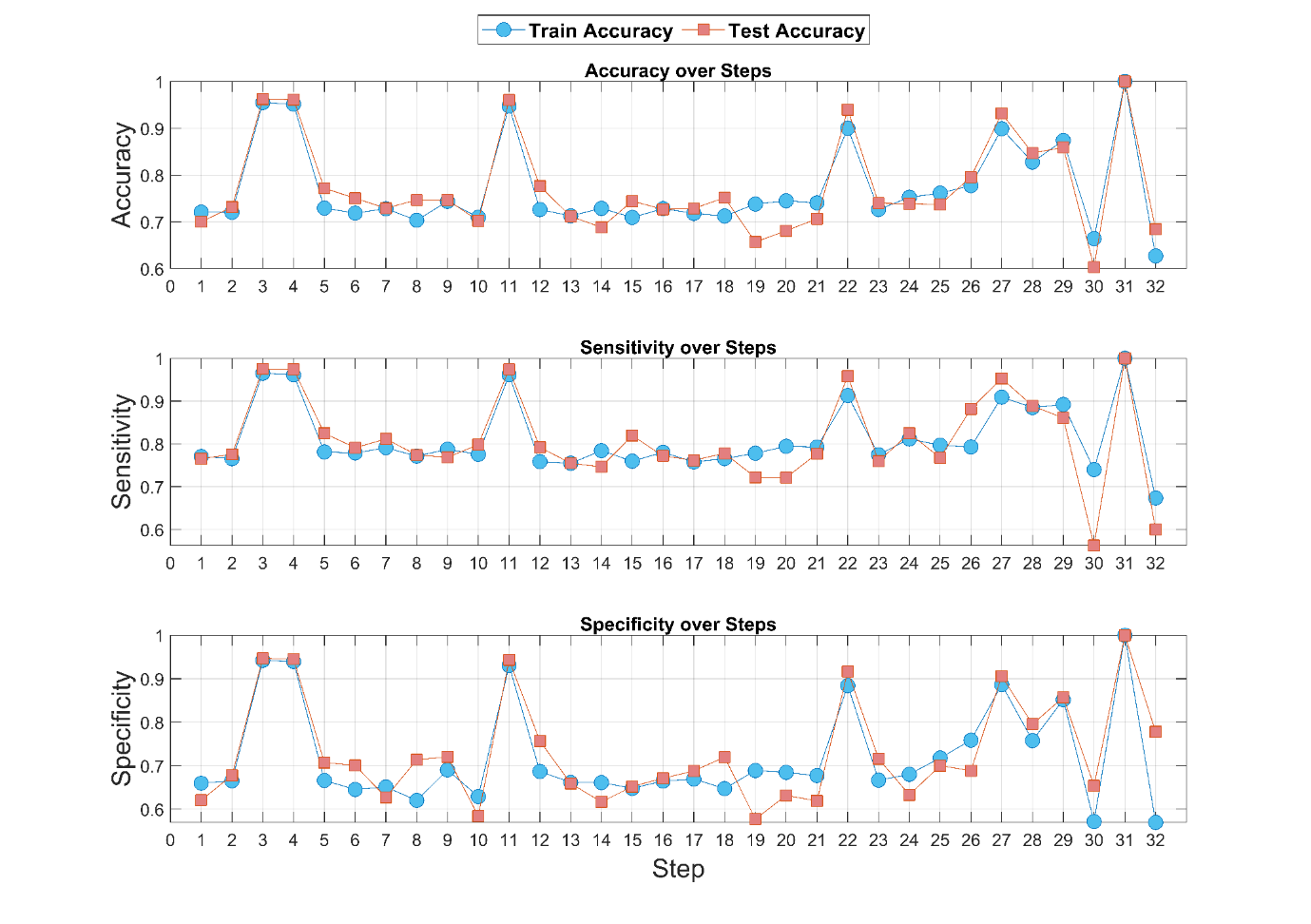


**Figure S1**. Evolution of validation (blue) and test (soft reddish-pink) performance metrics over 32 steps of sensor elimination for distinguishing between healthy individuals and ovarian cancer patients. The x-axis represents the step number, where at each step the sensor with the lowest similarity coefficient was removed from the array. The y-axis shows the percentage for accuracy (top panel), sensitivity (middle panel), and specificity (bottom panel).

**Section S1. Signal processing**

All signals were processed using the Savitzky-Golay smoothing filter, followed by data normalization to [0, 1] range. The normalization was applied across all samples within each class, not per sensor or within each batch. In our analysis, the Savitzky-Golay smoothing filter was applied using the MATLAB *smoothdata* function as follows: SS = smoothdata(RS, "sgolay"), where SS is smoothed signal, RS is the raw signal. The polynomial order used is 2, which is the default for the "sgolay" method and corresponds to a quadratic polynomial. The window size is automatically determined by MATLAB based on a heuristic that depends on the default smoothing factor, which in our case is 0.25. Specifically, MATLAB selects a window size such that approximately 25% of the high-frequency energy in the input signal is attenuated. The exact window size is data-dependent and computed internally by *smoothdata* based on the length and variability of the input data vector RS.

**Section S2. Sensor elimination algorithm**

1. In the first iteration, data from all 32 sensors were included. 85 features were extracted from each signal (including statistical, time-domain, and frequency-domain features), and training and test datasets were formed using a 90/10 split scheme. A comprehensive overview of the features is available in reference (23). The 90/10 train-test split was chosen to maximize the training data available for our Optimizable Ensemble model, given the relatively small dataset size and the complexity of the 85 extracted features, ensuring robust model learning while reserving a sufficient test set for evaluation. In other words, this split prioritizes model training in the context of high-dimensional feature space and limited samples. The split was stratified by class to maintain proportional representation of each class in both the training and test sets, preserving the class distribution. However, the split was not stratified by batch (KI batch 1, LiU batch 2/3), as the primary focus was on class-based performance rather than batch-specific effects, which were addressed through the use of both raw and smoothed-normalized signals in feature extraction. The Optimizable Ensemble model was trained (using a 5-fold cross validation scheme) and tested, generating performance metrics, including accuracy, specificity, and sensitivity at both validation and testing levels. The Optimizable Ensemble model was trained using MATLAB’s *fitcensemble* function with hyperparameter optimization performed via Bayesian optimization and the expected-improvement-plus acquisition function. The tuned hyperparameters included the ensemble method (e.g., Gentle Adaptive Boosting, Bootstrap Aggregation (Bagging) and Random Forest etc.), the number of learning cycles, the learning rate, and the minimum leaf size.

2. In the second iteration, the entire sequence was repeated, except that data from the least useful sensor (with the highest similarity coefficient) were excluded from the signal dataset.

3. In the third iteration, data from the two least useful sensors were excluded, and so on, up to the 32nd iteration, where data from only the most useful sensor remained.

**Section S3. Overfitting Considerations in Optimizable Ensemble Models**

We conducted a comprehensive set of analyses to explicitly test for overfitting and implemented strategies to mitigate it, ensuring the robustness and generalizability of our model. For this purpose, we utilized a feature dataset that excluded data from the two least informative sensors. We implemented a stratified 90-10 train-test split to maintain consistent class proportions across the training and test sets, as detailed in the revised manuscript. Initially, we defined a comprehensive hyperparameter search space for an ensemble classifier. This search space encompassed key parameters, including the ensemble method (AdaBoostM1, Bag, GentleBoost, LogitBoost, RUSBoost), number of trees (ranging from 10 to 500), learning rate (0.01 to 1), tree complexity parameters such as minimum leaf size (1 to 50) and maximum number of splits (1 to 50), split criterion (Gini’s Diversity Index or deviance), and the number of features to sample at each split (1 to the total number of features in the training data). Next, we performed hyperparameter optimization with 5-fold cross-validation, evaluating up to 30 combinations to identify the parameter set that minimized cross-validation error. After determining the optimal hyperparameters, we proceeded to train what we call an optimal ensemble model using these parameters, tailoring the configuration based on the selected ensemble method. To assess potential overfitting, we evaluated the model’s performance by computing the training error, cross-validation error, and test error on the test set. These error metrics are enabled to compare the model’s performance across training, validation, and test sets, ensuring robustness and generalization. The perfect training error (0%) suggests that the model has fit the training data extremely well, which can be considered as a potential indicator of overfitting. However, the relatively low cross-validation (3.38%) and test (4.72%) errors, computed using 5-fold cross-validation and the held-out test set, demonstrate good generalization, with only a small gap between training and validation. As the model generalizes well to unseen data, the risk of overfitting appears minimal. This suggests that the high diagnostic accuracy is likely driven by strong, biologically relevant signals in the dataset rather than excessive model optimization. However, to further confirm the robustness of our model and address the reviewer’s concern about potential overfitting, we implemented additional analyses, including model simplification, feature reduction, and constrained hyperparameters. These strategies were implemented to validate the model’s generalizability, improve interpretability, and confirm that performance was not overly reliant on specific model configurations or features. Specifically, the Simplified model used the optimal model’s hyperparameters (GentleBoost, MinLeafSize=50, MaxNumSplits=50) without further hyperparameter optimization, but reduced complexity by halving the number of learning cycles (from 499 to 250) and the learning rate (from 0.60382 to 0.30191). The Reduced model performed hyperparameter optimization on the top 50% of predictive features (selected via predictorImportance), resulting in adjusted parameters (e.g., LearnRate=0.98395, MinLeafSize=30, MaxNumSplits=41). The Constrained model conducted hyperparameter optimization within a restricted search space (e.g., NumLearningCycles=[50, 200], LearnRate=[0.01, 0.2], MinLeafSize=[20, 100], MaxNumSplits=[5, 30]), yielding parameters such as NumLearningCycles=185, LearnRate=0.19968, MinLeafSize=65, and MaxNumSplits=30, to prevent over-optimization and enhance generalization. These analyses, conducted across two random stratified 90-10 train-test splits, confirmed that the model’s high performance (Figures S2–S3) is robust across various configurations, as exemplified by the hyperparameters for the first split in Table S1. The small gap between cross-validation and test errors across all models in both splits, visualized in Figure S4, further supports minimal overfitting, reinforcing confidence in the model’s reliability for diagnostic applications.


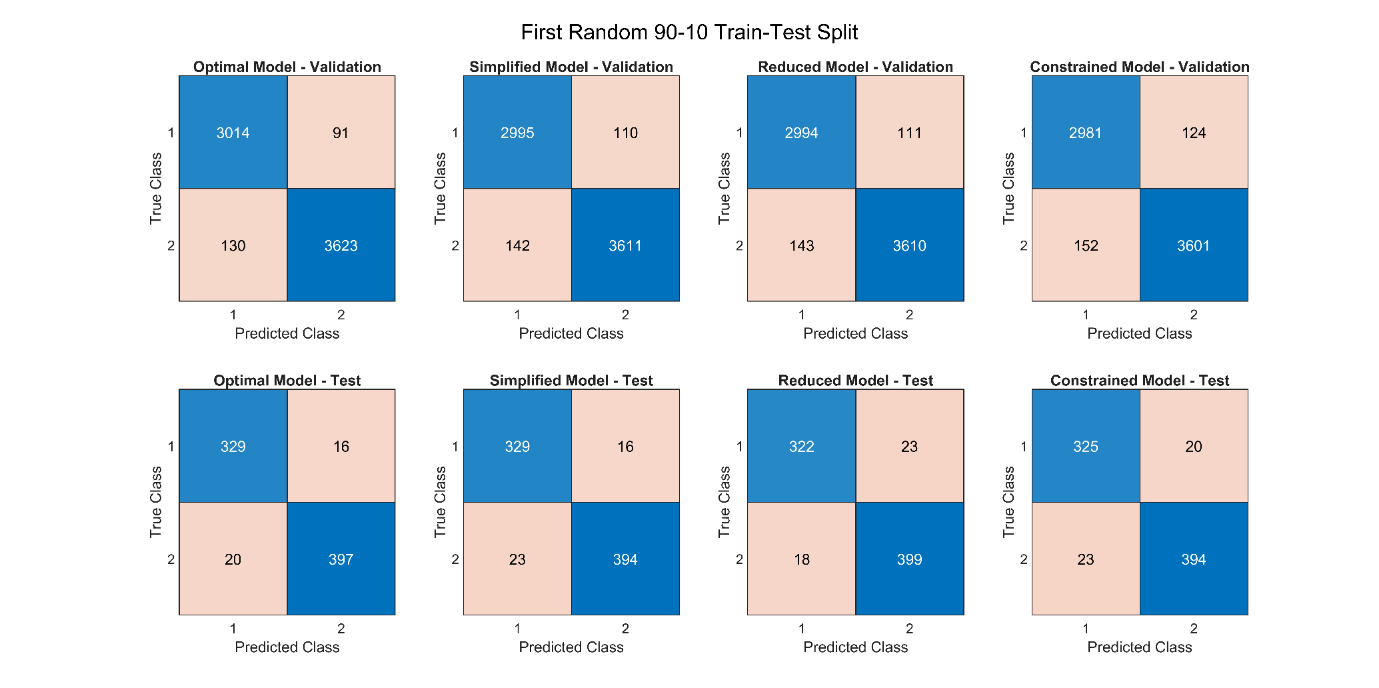


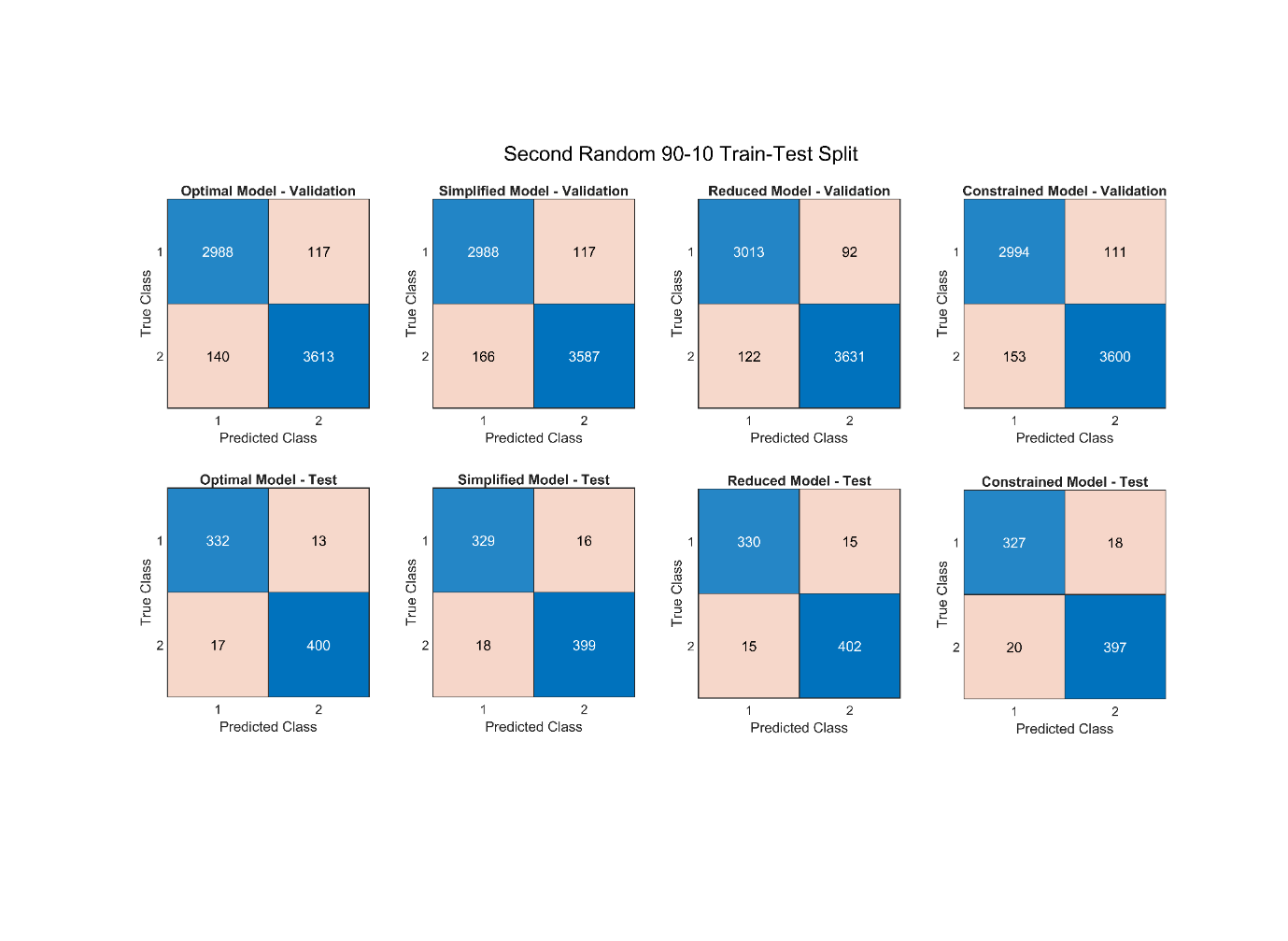


**Figure S2. Validation and test confusion matrices for all considered models.**


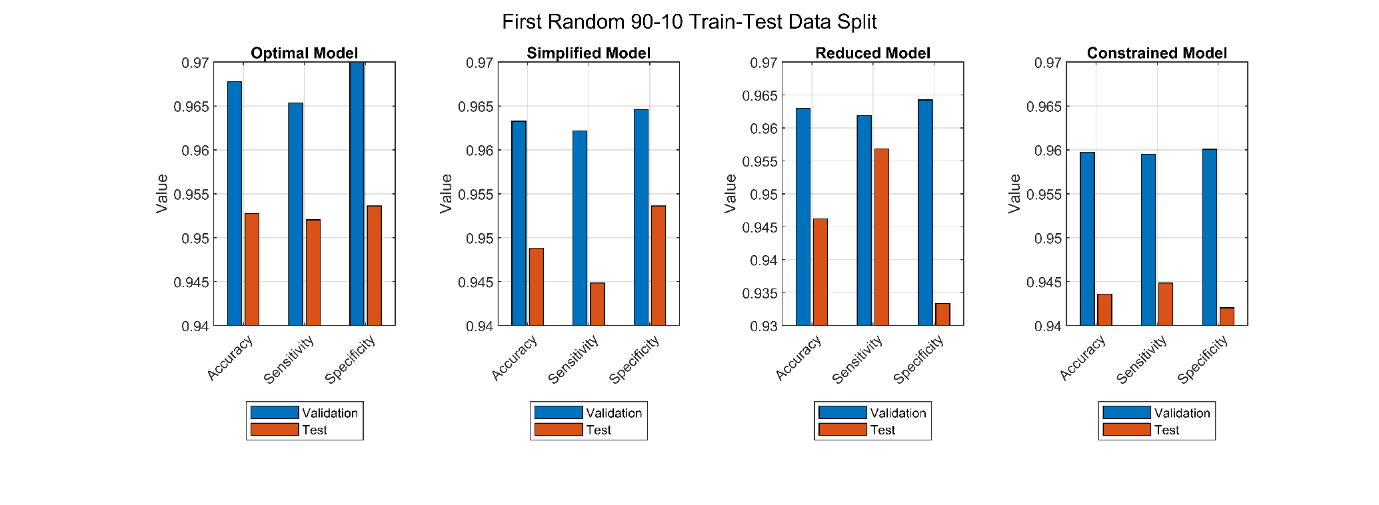


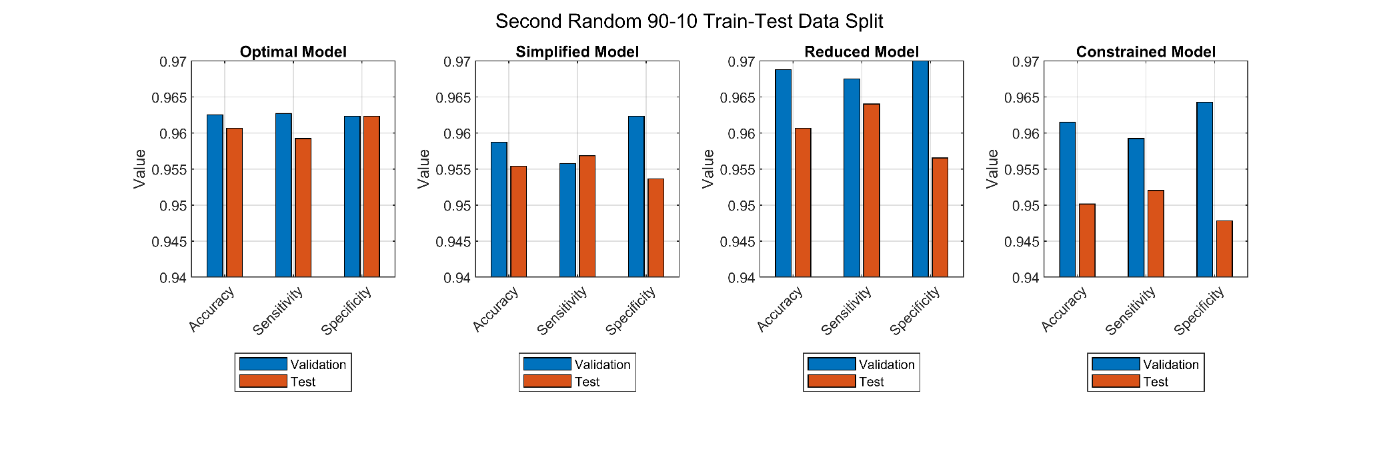


**Figure S3. Performance metrics for all considered models.**


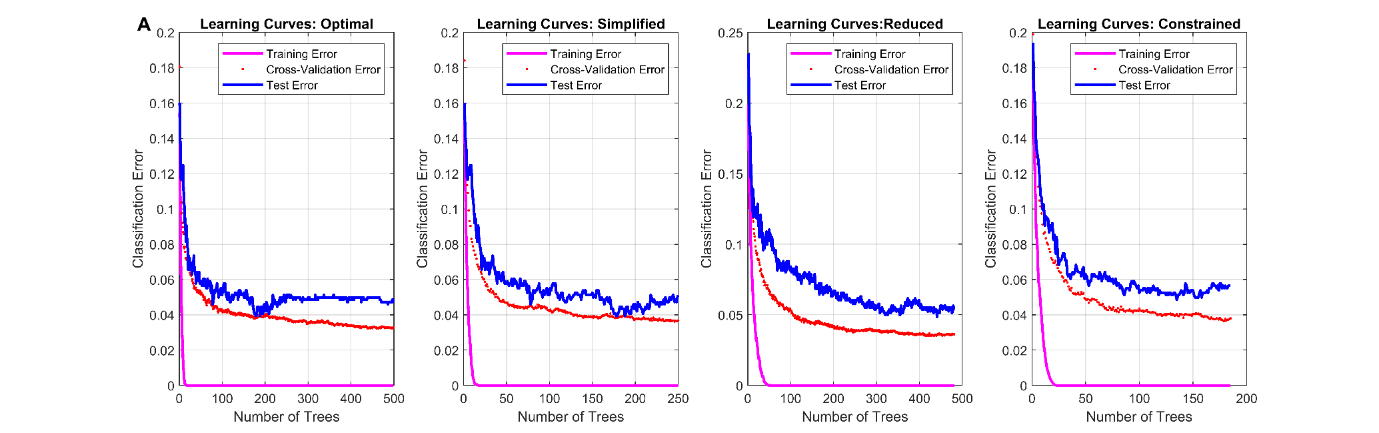


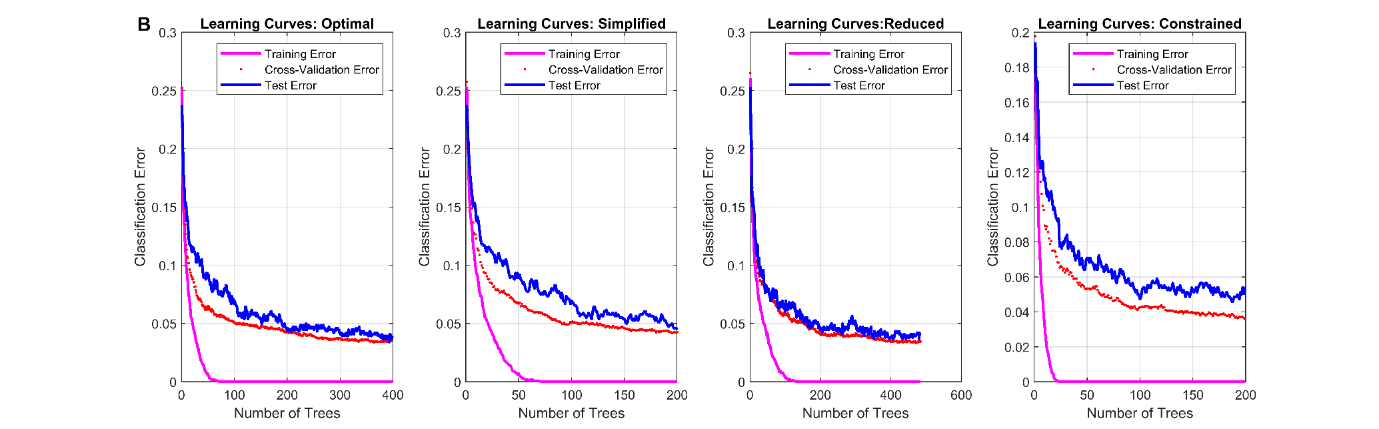


**Figure S4. Learning curves for ensemble models across two random train-test splits (A- first random 90-10 train-test split, B - second random 90-10 train-test split). The plots display training error (magenta), 5-fold cross-validation error (red), and test error (blue) as a function of the number of trees for the Optimal (subplot 1), Simplified (subplot 2), Reduced (subplot 3), and Constrained (subplot 4) models, demonstrating robust generalization with small gaps between cross-validation and test errors.**

**Table S1**. Hyperparameter configurations for ensemble models.

| **Model** | **Method** | **NumLearningCycles** | **LearnRate** | **MinLeafSize** | **MaxNumSplits** |
| --- | --- | --- | --- | --- | --- |
| Optimal | GentleBoost | 499 | 0.60382 | 50 | 50 |
| Simplified | GentleBoost | 250 | 0.3019 | 50 | 50 |
| Reduced | GentleBoost | 499 | 0.98395 | 30 | 41 |
| Constrained | GentleBoost | 185 | 0.19968 | 65 | 30 |
